# Mummy Induces Apoptosis Through Inhibiting of Epithelial-Mesenchymal Transition (EMT) in Human Breast Cancer Cells

**DOI:** 10.31661/gmj.v9i0.1812

**Published:** 2020-08-10

**Authors:** Solmaz Rahmani Barouji, Arman Shahabi, Mohammadali Torbati, Seyyed Mohammad Bagher Fazljou, Ahmad Yari Khosroushahi

**Affiliations:** ^1^Department of Persian Medicine, School of Traditional Medicine, Tabriz University of Medical Sciences, Tabriz, Iran; ^2^Student Research Committee, Tabriz University of Medical Sciences, Tabriz, Iran; ^3^Molecular Medicine Department, Faculty of Advanced Medical Sciences, University of Medical Sciences, Tabriz, Iran; ^4^Department of Food Science and Technology, Faculty of Nutrition and Food Sciences, Tabriz University of Medical Sciences, Tabriz, Iran; ^5^Drug Applied Research Center, Tabriz University of Medical Sciences, Tabriz, Iran; ^6^Department of Medical Nanotechnology, Faculty of Advanced Medical Science, Tabriz University of Medical Sciences, Tabriz, Iran

**Keywords:** Mummy, EMT, TGFβ1, Apoptosis, Breast Cancer

## Abstract

**Background:**

Mummy (Iranian pure shilajit) is a remedy with possessing anti-inflammatory, antioxidant and anticancer activities. This study aimed to examine mummy effects on epithelial-mesenchymal transition (EMT) and invasiveness of MCF-7 and MDA-MB-231 breast cancer (BC) cell lines with underlying its mechanism.

**Materials and Methods:**

The dose-dependent inhibitory effect of the mummy on cell proliferation *in vitro* was determined using the MTT assay. Flow cytometry and 4’,6-diamidino-2-phenylindole dihydrochloride staining were respectively used for quantitative and qualitative analysis of cellular apoptosis, and gene expression analysis was conducted using real-time PCR.

**Results:**

MDA-MB-231 showed more sensitivity than the MCF-7 cell line to the anticancer activity of mummy, while mummy did not exhibit significant cell cytotoxicity against human normal cells (MCF-10A). The gene expression profile demonstrated a significant decrease in TGF-β1, TGF-βR1, TWIST1, NOTCH1, CTNNB1, SRC along with an increase in E-cadherin mRNA levels in mummy treated cells compared to the untreated control group (P≤0.05).

**Conclusion:**

Mummy triggers inhibition of EMT and metastasis in breast cancer cells mainly through the downregulation of TGFβ1 activity, and more studies required to find its specific anticancer activity with details.

## Introduction

**Figure 1:**
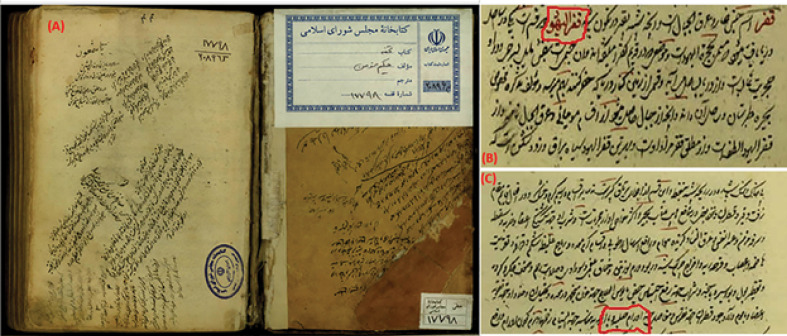
The ancestor document that represents the mummy therapeutic effects on cancer treatment. (A) represents the old handwritten book named Tohfeh, and panels (B) and (C) illustrate the text in the Tohfeh book that shows the mummy and cancer meaning into the red box, respectively.

Breast cancer (BC) is the predominant life-threatening cancer among females worldwide [1,2]. Despite remarkable progress in the development of chemotherapeutic anticancer drugs, cancer death rates on to rising [3,4]. In advanced BC, chemotherapy has been the only mainstay treatment [5] hence the molecular mechanism of BC must be discovered and new potential anticancer drugs should be administered. Traditional Medicine (TM) has been used as a promising complementary strategy for serious disorders including cancer [6-8] and mummy (commonly known as Shilajit) has long been used in TM as a blackish-brown herbal compound [9]. Based on our ancestors documents, the mummy has been used for the treatment of all kind of cancers and we find this issue in old handwritten book “Tohfeh” and the mentioned document is presented in Figure-1. According to previous reports, the efficacy of mummy associated with humic and fulvic acids, which are essential for its biochemical functions that these compounds represented to be very effective in decreasing inflammation, pain, arthritis, ulcer, diabetes, stress, anxiety, and the most importantly inducing the cell apoptosis in many cancers. Mummy, as a water-soluble non-toxic and inexpensive compound, can be consumed as a part of the daily diet [10] thus, further studies required to investigate the therapeutic effects of mummy and its active constituents as suitable candidates for chemotherapy. Although some reports have indicated the anticancer effects of the mummy in cancer types including BC [10-12], however, the certain effects and cell signaling pathways related to its anticancer activity against BC cells are not fully identified. Therefore, this study aimed to determine the roles of the mummy in controlling the epithelial-mesenchymal transition (EMT), and induction of apoptosis in MCF-7 and MDA-MB-231 cancerous cells and to ascertain the potential molecular mechanisms involved in BC. Overall, EMT has been recognized as a key process of cellular remodeling during embryogenesis (Type 1 EMT), regeneration of tissue and fibrosis program (Type 2 EMT) and closely associated with reinforcing drug resistance, metastasis and cancer progression (Type 3 EMT) [13,14]. EMT can be regulated through several growth factors and signaling pathways, including transforming growth factor-β1 (TGF-β1), Hepatocyte growth factor (HGF), fibroblast growth factor (FGF), Wnt and Notch pathways [15,16]. TGFβ1 is considered as a multifunctional cytokine since TGF-β1 has been reported as a master inducer of EMT in both physiological such as fetal development, regulation of cell proliferation and differentiation, scar formation and fibrosis induction and pathological situations such as angiogenesis migratory phenotype and cancer progression (EMT type 3) [17]. One important aspect of TGF-β1 function is its interaction with many factors, including EMT-associated transcriptional regulators, such as TWIST1 [18] and CTNNB1 [19], EMT markers like E-cadherin [20], other oncogenic signaling pathways such as NOTCH1 [21], and Smad-binding elements like SRC [22]. In consequence, understanding the crosstalk between the TGF-β1 signaling and these agents may provide deeper insight into the development of novel therapeutic strategies for inhibition TGF-β1-induced EMT and cancer therapy. This study aimed to exam the anti-cancer activity of mummy against non-metastatic BC cell line MCF-7 and metastatic MDA-MB-231 cell line in vitro to provide groundbreaking for the treatment of BC by mummy. For evaluating mechanisms involved in mummy anticancer effects, the expression of genes involved in EMT phenomena such as TGF-β1, TGF-βR1, TWIST1, CTNNB1, NOTCH1, and SRC were assessed in MDA-MB-231 and MCF-7 BC cells.

## Materials and Methods

###  Materials

**Figure 2:**
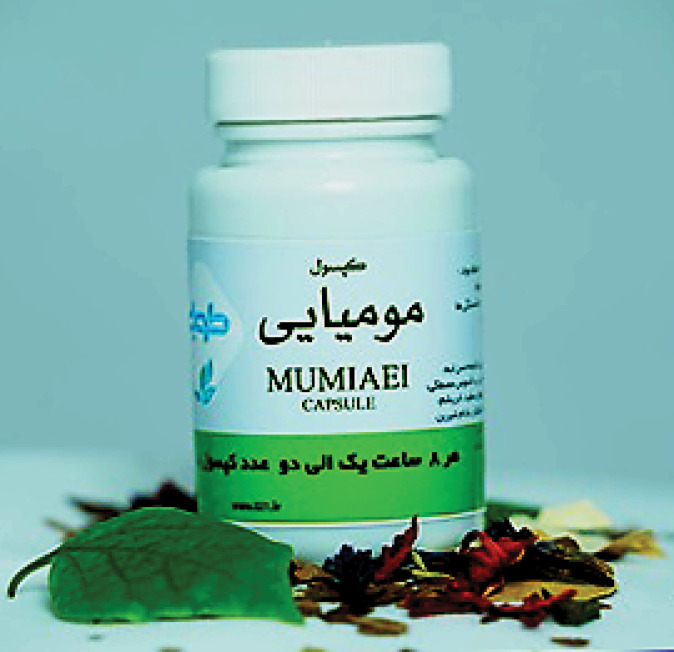
The pharmaceutical grade of Iranian mummy used for performing this research, batch number; 92-0199-s

Water-soluble pharmaceutical grade of the mummy was purchased from Tuba company (http://tuba18.ir/fa/, Tehran Iran, batch number; 92-0199-s) as traditional medicine product and Figure-2 shows the product pharmaceutical packaging that illustrates the drug license from Iranian ministry of health and medical education. Roswell Park Memorial Institute (RPMI) 1640, trypsin and ethylenediaminetetraaceticacid (EDTA), 3-(4,5-dimethylthiazol-2-yl)-2,5-diphenyltetrazolium-bromide (MTT) solution (Sigma-Aldrich, St. Louis, USA), phosphate-buffered saline (PBS), Fetal bovine serum (FBS), 4’,6-Diamidino-2-Phenylindole, Dihydrochloride (DAPI) were obtained from Sigma Co. (St. Louis, MO, USA). Annexin V-fluorescein isothiocyanate (FITC), propidium iodide (PI) were purchased from E-bioscience (eBioscience, San Diego, CA). Cinagene Kit (RNX-Plus Solution, SinaClon, Iran), SYBR Green PCR master mix (Takara Bio Inc., Tokyo, Japan), and primers were obtained from Takapouzist Co. (Tehran, Iran). The MDA-MB-231 and MCF-7 human breast cancer cell lines and MCF-10A normal mammary epithelial cells were procured from National Cell Bank, (Pasteur Institute, Tehran, Iran).

###  MTT Assay 

All cancerous and normal cells with seeding density (104^4^cells/cm^2^) were cultured in RPMI medium supplemented with 10 % FBS and incubated at 37 °C in 5% CO2. The overnight cultured cells medium were replaced with fresh media containing 10, 20, 30, 40, 50, 60, 70, 80, 90 and 100 µg/ml mummy as treated groups and untreated cells also cells treated with cisplatin were considered as negative and positive control groups, respectively. For MTT assay, the previous medium of cells was replaced with 200 μl fresh growth medium containing 20 μl of MTT solution (2 mg/ml) and incubated for 4 h at 37˚C. The MTT containing media was removed after 4 h then, 200 μl of DMSO was added to each well for dissolving the formazan crystals, and the absorbance was measured at 570 nm using a microplate reader (Biotek, ELx 800, USA).

###  The Apoptotic Assay Using DAPI Staining

The morphological assessment of apoptotic cells was characterized using a DAPI nuclear staining method [23]. Cells at a density of 4×10^5^ (cells/well) were seeded in six-well culture plates. The over nightly cultured cells were treated with the mummy at IC50 concentration for 24 h then cells were washed with PBS (5 ml) and were fixed using paraformaldehyde (4%) for 20 min. The cells were permeabilized using 0.1 % (w/v) Triton X- 100 and were stained with DAPI stain solution (250 ng/ml for each well) for 15 min. Nuclear morphology alteration was evaluated using fluorescent microscopy (Olympus BX64, Olympus, Japan).

###  Quantitative Apoptosis Assay Using Flow Cytometry 

To determine the percentage of cell apoptosis and discrimination them from the necrotic phase, cells were quantitatively analyzed by flow cytometry using the FITC-Annexin V apoptosis detection kit [24]. Briefly, cells (4×10^5^ cells/well) were treated with the IC50 concentration of mummy at 37°C for 24 h. Collected cells from each well were washed with PBS and then incubated with Annexin V-FITC and propidium iodide (PI) in binding buffer for 15 min at dark condition based on kit instruction. Stained cells were analyzed using flow cytometry using 150000 cells at a rate of 900 cells/s. Data analysis was conducted using CELL Quest Pro software (BD Biosciences, San Jose, CA, USA) and quadrant settings were fixed with untreated, single-stained controls and copied to dot plots of the treated cells.

###  RNA Isolation, cDNA Synthesis, and RT-PCR

Total RNA was extracted from the cells using the Cinagene Kit (RNX-Plus Solution, SinaClon, Iran) according to the manufacturer’s recommendations [25]. cDNA was produced by using a synthase kit (Qiagen) following the manufacturer’s data. Table-1 list of primer sequences designed for amplifying intended genes. Each qPCR reactions were accomplished triplicate for all sample, containing 10 μl SYBR Green PCR master mix, 1μl cDNA (1 μg/μl), 1 μl primer (forward and reverse) and 0.8 μl 6-carboxy-X-rhodamine (ROX as reference dye) using ABI-step I plus (Applied Biosystems, Forster City, CA, USA) instrument and negative controls were included in each experiment. The following cycling condition was performed using a protocol: 40 cycles at 95ºC for 20sec, annealing temperature for 35 sec, and at 72ºC for 10 sec. The quantitative real-time PCR data were analyzed using 2^-δδCT^ values were normalized to the expression rate of GAPDH as a housekeeping gene.

###  Statistical Analysis

 Data were presented as the mean ± SD of three independent experiments. One-way ANOVA was performed to determine significant differences among all examination groups. The statistical analysis was conducted by using the statistical package for the social sciences (SPSS Inc. Chicago, IL, USA version 16.0). P-value≤0.05 was considered a significant difference (Graph Pad V6.0 Software Inc., San Diego, CA, USA).

## Results

###  Inhibitory Effect of the Mummy on Cell Growth 

**Figure 3:**
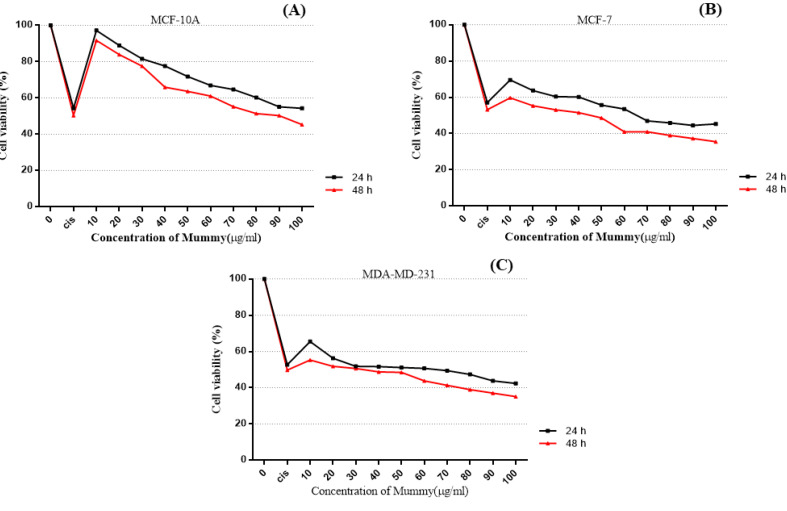
Cytotoxicity effect of various concentrations of the mummy in human breast cancer and normal cell lines.

 To evaluated the in vitro anti-tumor effects of the mummy, we included a very metastatic variant of BC cell-line, MDA-MB-231, low-metastatic cell line, MCF-7, and normal mammary epithelial cells, MCF10A. All three cell lines were incubated to various concentrations of mummy and cell viability was detected by MTT assays. The IC50 values for mummy treated MCF-7 and MDA-MB-231 were obtained 40±0.8µg/ml and 31.3±0.4 µg/ml, respectively, while the IC50 of MCF10A cells was 89.66±1.2 µg/ml that indicate resistance to cell death by mummy compared to cancerous cells. As shown in Figure-3, a dose-dependent cell-growth inhibition was observed in mummy-treated MCF-7 and MDA-MB-231 cells. Besides, the inhibitory effect of the mummy at the same dose on cell growth of MCF7, MDA-MB-231, and MCF10A normal cells was increased by an increase in the incubation time (Figure-3).

###  Effect of the Mummy on the Apoptosis 

**Figure 4:**
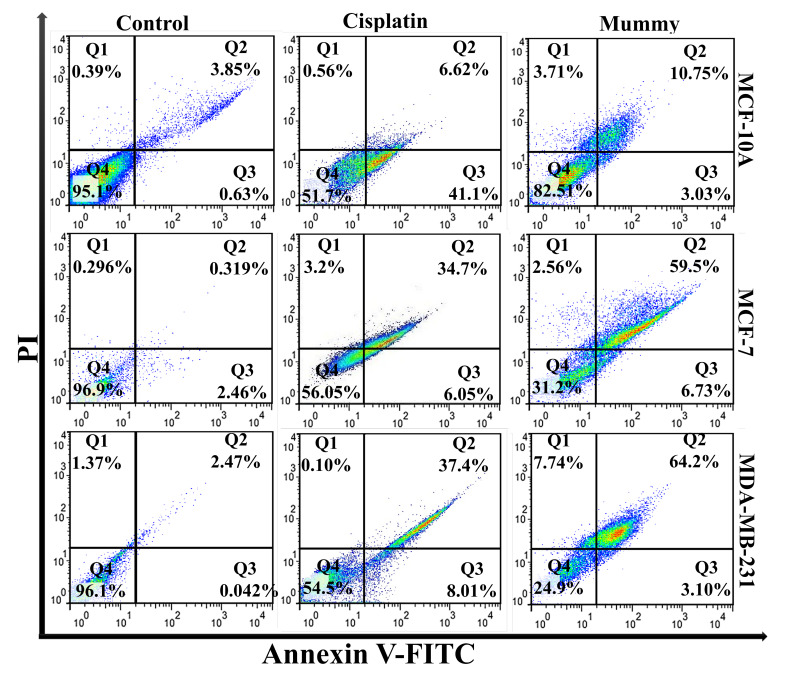
Flow cytometry analysis of untreated (control) and treated tumor and normal cell lines. 4 distinct phenotypes: Q1 (FITC/PI -/+; Q1) as necrotic cells; Q2 (FITC/PI +/+; Q2) as late apoptosis cells; Q3 (FITC/PI +/-) as early apoptotic cells and Q4 (FITC/PI -/-) as viable cells.

**Figure 5:**
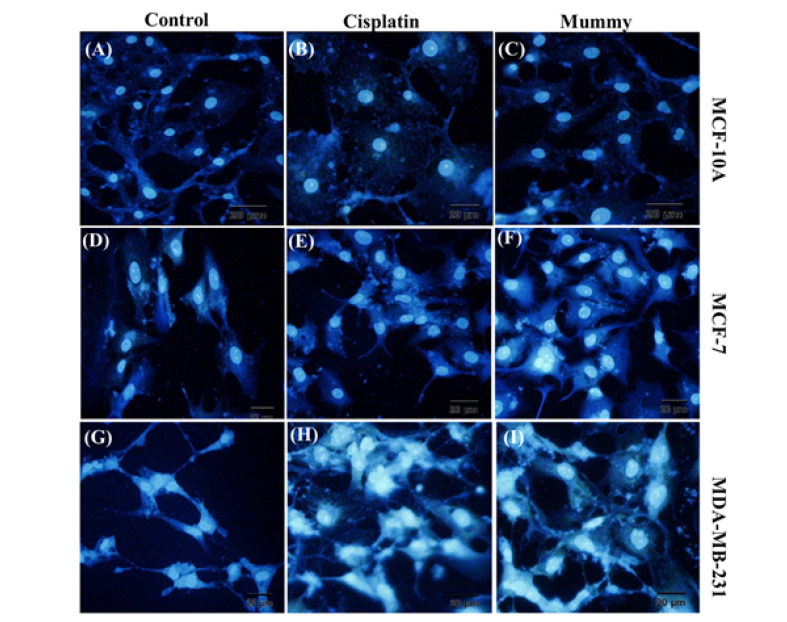
DAPI staining of nuclear morphological changes induced by mummy in breast cancer and normal cell lines.

 To determine whether cultured BC cells undergo apoptosis, untreated or mummy-treated MCF-7, and MDA-MB-231 cells were analyzed with Annexin-V and PI. Mummy- treated MDA-MB-231 and MCF-7 cells showed 1.4-fold and 1.6-fold higher the percentage of apoptosis than the cisplatin-treated group, respectively. Taken together, the percentage of apoptotic cells increased in mummy-treated MDA-MB-231 compared to mummy-treated MCF-7cells (67.3±2.17 vs. 59.83±1.57%, n=3, P≤0.05, Figure-4) by showing less than 10% of the population with necrotic signs in both of cell lines. In the mummy-treated MCF-10A group, the cell viability was only decreased from 95.1% to 82.53, indicating the mummy inhibition efficiency on the growth of BC cells with low cytotoxic effects on normal breast cells. To qualify the cell death mediated by apoptosis or necrosis, the DAPI staining method was used to assess the morphology of cell nuclei in untreated control or treated cells. DAPI staining confirmed our result of quantitative apoptosis analysis where the maximum rate of cell death (nuclear deformation and loss of cell wall integrity) was in the cells treated with mummy in MDA-MB-231 cancer cells (Figure-5 I).

###  Expression Analysis of Genes Involved in EMT

**Figure 6:**
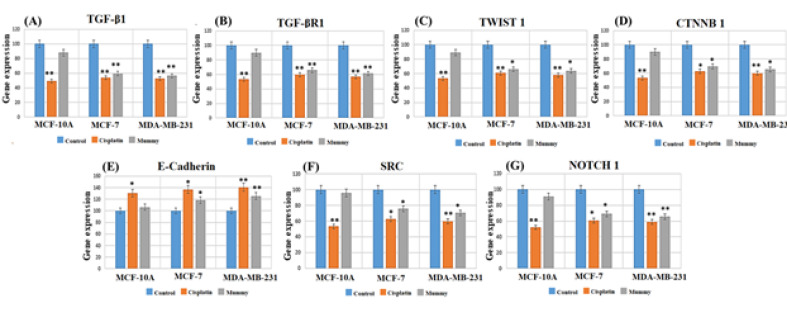
Effects of the mummy on the mRNA expression pattern of TGFβ-1, TGF-βR1, TWIST1, CTNNB1, NOTCH1, SRC, and E-cadherin genes in MCF-7, MDA-MB-231, and MCF10A cell lines. All data were normalized with GAPDH as reference genes. *P≤0.05, **P≤0.01.

 To explore the possible molecular mechanism underlying the effects of mummy against BC cells, the expression of the important gene (TGF-β1) was evaluated in the EMT pathway through real-time PCR (Figure-6). As shown in Figure-6A, the expression ratio of TGF-β1 in all untreated groups was significantly higher in MDA-MB-231 as well as MCF-7 than the MCF10A cells. Mummy treated MDA-MB-231 and MCF-7 cells showed a significant decrease in TGFβ-1 expression compared to their related control groups while no significant change was observed in mummy treated MCF-10A cells as compared to its related untreated control group. Using the mummy caused a significant decrease in TGFBR1 mRNA expression level in both BC cells (P≤0.05). To more thoroughly verify the effect of the mummy on EMT, we focused on the EMT nuclear transcription factors TWIST1 and CTNNB1 and epithelial markers (E-cadherin). Based on our findings, mummy decreased the mRNA expression level of TWIST1 and CTNNB1 in MCF-7 and MDA-MB-231 cells (Figure-6C and 6D), while it increased mRNA expression levels of E-cadherin (Figure-6E). The decreased expression level of NOTCH1 and SRC genes, EMT inducing and cell invasion promoting genes in BC was also observed in both BC mummy treated cell lines (Figure-6F and 6G). Based on this study findings, the mummy was able to reverse the EMT process and induces apoptosis in BC cells, where these processes were not observed in MCF10A breast normal cells.

## Discussion

Since metastasis is still an important obstacle in the BC, various chemotherapeutics have been used for BC therapy to improve the survival rate and control of tumor growth in BC patients. TM has been used for cancer study and numerous traditional herbal remedies have been revealed to prevention and cancer chemotherapy [26]. Among them, mummy is one of the medicinal compounds used traditionally in cancer cures. The reduction of cancerous cell proliferation and the induction of cancerous cell apoptosis is the main therapeutic strategies in cancer treatment [27]. Based on previous studies reports, mummy possesses the capability for inducing apoptosis as well as suppress proliferation in several kinds of cancerous cells [28,29]. This study finding disclosed the effect of different concentrations of mummy on cell growth of BC MCF-7 and MDA-MB-231 cells was evaluated using an MTT assay. Our finding illustrated that mummy inhibited BC cell viability, and the rate of mummy-induced cellular proliferation inhibition dramatically increased even at low concentration (0.01 mg/ml) (P<0.05). Following the staining of the cells with Annexin V-FITC and PI, cell apoptosis was examined by using flow cytometry. Data revealed that mummy enhanced the percentage of BC cell apoptosis. Additionally, DAPI staining demonstrated that mummy could increase the apoptosis of MCF-7/MDA-MB-231 BC cells. By considering defects in apoptotic signaling contribution in many malignant tumors [30], the anti-cancer activity of mummy through inducing apoptosis in cancerous cells with lower impacts on normal cells can be raised its main important role at least in breast cancer therapy. Types of BC cell lines with varying degrees of differentiation and invasiveness have been obtained from clinical patients, and some of them show EMT characteristics, which are down-regulation of epithelial biomarkers and up-regulation of mesenchymal biomarkers. For instance, the MDA-MB-231 cell line has been shown the decreased expression of E-cadherin and more invasiveness than the MDA-MB-468 cell line [31]. Based on our findings, both BC cell lines (especially MDA-MB-231) expressed high levels of genes involved in the EMT process thus we hypothesized the mummy anticancer activity in previous studies [10,32], may be related to down-regulation of these genes and reversing of EMT in BC. This study is the first to investigate the apoptotic and anti-Metastatic effect of mummy on BC cells by suppressing EMT in vitro. Previous studies obtained evidence that in BC patients, aberrant TGF-β1 expression was correlated with worse survival [33]. Overexpression of TGF-β1 has an aggressive phenotype, including promoting cell proliferation, invasion, and stem cell properties, which suggested that TGF-β1 might be a potential marker for cancer diagnosis and therapy. For example, Vitiello GA showed that TGF-β1 signaling plays an important role in the EMT process and has been reported to stimulate BC cells to undergo EMT [34]. Since changes in EMT are often accompanied by increased expression of TGF-β1 [35], the TGF-β1 level determined after mummy treatment and it is found that mummy also effectively inhibited the expression of TGF-β1 in MCF-7/MDA-MB-231cells (P≤0.05). Furthermore, Castillejo *et al* reported that the suppression or overexpression of TGF-β1 signaling leads to disruptions in TGF-βR1 or TGF-βR2 which are related to increased cancer risk [36]. Similarly, we observed a differential expression level of TGF-βR1 among diverse BC cells apart from MCF10A. The expression of TGF-βR1 are usually down-regulated following down-regulation of TGF-β1, this relationship is also observed in this study. Various *in vivo* and *in vitro* studies have discovered that TGF-β1 contributes to BC initiation and development through the activation of transcription factors such as TWIST which play important roles in the EMT process [37,38]. Of note, The induction of TWIST1 mRNA during EMT may be reversed by the inhibition of TGF-β1 signaling [39]. More recently, few studies revealed the direct and indirect role of TWIST1 in the inhibition of a tumor suppressor E-cadherin [40]. It is well known that inhibition of E-Cadherin will cause an accumulation of CTNNB1 within cells leading to nuclear translocation where the abnormal accumulation of CTNNB1 is significantly associated with many cancers [41]. The results agreed with our expectations that a decrease in TWIST1 expression in mummy treated metastatic and non-metastatic BC cells lead to a reduction of CTNNB1 and induction of E-cadherin. However, the efficacy of mummy to down-regulate the expression of TWIST1 independent of TGF-β1 cannot be neglected. Zhang H *et al* found that Src, a molecule associated with EMT, mediates many of the processes involved in the ability to invade and disseminate of the tumor cells [42], which activated TGF-β1 [43]. This study results reviled the decrease Src expression in mummy treated BC cells may be dependent on decreasing TGF-β1 or directly by mummy. Besides, NOTCH1 is highly expressed in BC cells in clinical and experimental models [43,44] thus to more thoroughly verify the effect of the mummy on EMT, an investigation of the NOTCH1 expression levels is performed. The inhibitory effect on NOTCH1 gene expression in BC cells treated with mummy was also observed in both MCF-7/MDA-MB-231 BC cell lines. By considering TGF-β axis dysregulation as a conserved driver of EMT phenotype in several models of BC, this study suggested that TGF-β1 might be an upstream factor regulating EMT and thereby plays an important role in EMT mesenchymal phenotype. Upon this study’s findings, mummy as a traditional remedy has shown proliferation inhibition effects on breast cancer cell lines and without significant negative effects on normal epithelial cells. One of the mechanisms for anticancer effects of the mummy is related to its antiproliferative activity thus we proposed the investigation of effects of mummy on the different anticancer pathways such as the down-regulation of ErbB family genes.

## Conclusion

Mummy has profound activity against MCF-7 and MDA-MB-231 BC cell lines but at the same concentration, mummy only caused a little damage to normal cells (MCF-10A cell). This study collective data suggest that mummy inhibits cell growth of MCF-7 and MDA-MB-231 BC cells through inhibition EMT and induction apoptosis. However, we cannot rule out the possibility that mummy may also act through other mechanisms to inhibit EMT, and metastasis in BC cells. Our findings are based on *in vitro* study and clinical use of this substance needs further research *in vivo* is necessary to clarify the specific anti-cancer mechanism of the mummy, which could contribute to the development of novel mummy-related drugs.

## Acknowledgment

 The financial support of the Tabriz University of Medical Sciences is gratefully acknowledged. The results of this article are derived from the PhD thesis of Solmaz Rahmani Barouji registered in Tabriz University of Medical Sciences, Tabriz, Iran.

## Author Contribution

 Solmaz Rahmani Barouji and Ahmad Yari Khosroushahi designed and performed the study. Arman Shahabi carried out the cellular/molecular-based experiments. Solmaz Rahmani Barouji drafted the manuscript. Mohammadali Torbati revised the manuscript. Seyyed Mohammad Bagher Fazljou consults regarding traditional medicine. Ahmad Yari Khosroushahi edited and approved the final version of the manuscript. All authors have read and approved the final manuscript.

## Conflict of Interest

 All authors declare no potential conflicts of interest.

**Table 1:** The Primer Used for Quantitative Real-Time PCR

**Objective Genes**	**Primer Sequence**
**TGFβ1**	Forward: 5′-TATCGACATGGAGCTGGTGA-3′ Reverse: 5′-CCTCCTTGGCGTAGTAGTCG-3′
**TGFβR1**	Forward: 5′-GATGGGCTCTGCTTTGTCTC-3′ Reverse: 5′-CAAGGCCAGGTGATGACTTT-3′
**TWIST1**	Forward: 5′-AGTCCGCAGTCTTACGAGGA-3′ Reverse: 5′-CCAGCTTGAGGGTCTGAATC-3′
**E-cadherin**	Forward: 5′-GCGAACTGTTTGCAGAGG-3′ Reverse: 5′-CAGTGCGTGTCGTGGAGT-3′
**CTNNB1**	Forward: 5′-TCATGCGTTCTCCTCAGATG-3′ Reverse: 5′-AATCCACTGGTGAACCAAGC-3′
**SRC**	Forward: 5′-GGCTACATCCCCAGCAACTA-3′ Reverse: 5′-TGAGAGGCAGTAGGCACCTT-3′
**NOTCH1**	Forward: 5′-TCACGCTGACGGAGTACAAG-3′ Reverse: 5′-CCACACTCGTTGACATCCTG-3′
**GAPDH**	Forward: 5′-TGTGGGCATCAATGGATTTGG-3′ Reverse: 5′-ACACCATGTATTCCGGGTCAAT-3′

**Figure-1** documents translation

The nature of the **mummy** is something like bitumen coming out of the crevices of some of the mountains, most prominently in the Darab Mountains of the Fars province in Iran and later in the Mountains of Estahban and Kohgiluyeh in Iran.
 There are many drugs in the history of pharmacology that are classified into three categories as herbal, animal, and mineral. One of the types of mineral medicines widely used by physicians from the ancient to the late middle ages to treat various diseases is “mummy”. In the first century AD, Dioscorides found medicinal mummies from the coast of Apollonia useful for epilepsy and inflammatory and sciatica diseases (Al-Hashayesh of Dioscorides, p.173). Claudius Galenus also found mummies to be useful in skin diseases. In al-Nabat’s book of Dinavari and Ferdows al-Hikmah of Tabari, p.333 and p.405 respectively, mummies are useful for the treatment of fractures and wound healing. Razi, a famous Persian physician, wrote an independent book on the mummy subject and repeatedly used mummies to treat his patients. He had prescribed mummies for trauma, pain relief, fractures, injuries, headaches, tonic, brain tonic, earache, asthma, spleen pain and pediatric seizures (The medical treatises of Razi Pages 6 to 12, al-Hawi p. 82, al-Mansouri al-teb, p.177) In the tenth century, Ahwazi in the book of Kamel al-Sana’e considered mummy to be beneficial for cold headache, hemoptysis, asthma, and withdrawal of the dead fetus (Vol. 3, p. 206). Avicenna, a famous Persian physician, in his famous book, Qanoon found mummies very effective in brain tonic, fertility, and many other diseases. (Vol. 2, p. 17) In the 12th century, a Persian book called Zakhire Khwarazmshahi was written by Jorjani, a renowned Persian physician, who found mummies to be very useful for inflammation, ulcers, urinary problems, and prostates (p. 624, p. 815, p. 818).
Ibn Nafis, known in the thirteenth century for his discovery of blood circulation, considered mummies to be beneficial for cancers. A book by al-Adawiyah, written by Aghili Khorasani in the 16th century, one of the most famous books on pharmacology in Persian medicine, mentioned the treatment of **various types of tumors**, both benign and malignant, by mummies.
 In the 14th-century, in the book of Hakim Momen, mummies are useful for kidney stones and bladder, amenorrhea, and cataract treatment. The use of mummies to the bite of insects, snakes, and scorpions, sciatica pain, benign and malignant tumors have also been found useful. Mummy is even recommended for the treatment of some skin diseases such as psoriasis.
